# Independent preoperative predictors of day-of-surgery red cell transfusion in major orthopedic surgery: a six-year retrospective cohort of 7072 patients

**DOI:** 10.1016/j.bjane.2025.844685

**Published:** 2025-10-11

**Authors:** José R. Ortiz-Gómez, Andrea Ortiz-Domínguez, Inocencia Fornet-Ruíz, Francisco J. Palacio-Abizanda

**Affiliations:** a University Hospital of Navarre, Service of Anesthesiology (Section D), Pamplona, Spain; b Jiménez Díaz Foundation University Hospital, Service of Immunology, Madrid, Spain; c University Hospital Puerta de Hierro Majadahonda, Service of Anesthesiology, Madrid, Spain; d HM San Chinarro, Service of Anesthesiology, Madrid, Spain

**Keywords:** Anesthesia, Blood transfusion, Orthopedic surgery

## Abstract

**Background:**

Major Orthopedic Surgery (MOS) is frequently associated with significant blood loss, potentially resulting in perioperative anemia and the need for allogeneic blood transfusion, which carries inherent risks. This study aimed to identify independent preoperative predictors of early Packed Red Blood Cell (PRBC) transfusion in patients undergoing MOS.

**Methods:**

We analyzed, retrospectively, data from 7072 patients who underwent MOS. The variables assessed included age, sex, weight, height, Body Mass Index (BMI), ASA (American Society of Anesthesiologists) physical status classification, surgical category (hip, knee, spine), type of procedure (primary or revision total hip/knee arthroplasty, spinal arthrodesis, scoliosis surgery), preoperative hemoglobin levels and levels at 8:00 AM on postoperative day 1, hemoglobin thresholds (> 13, < 13, < 12, < 11, and < 10 g.dL^-1^), administration of tranexamic acid, and the requirement for PRBC transfusion.

**Results:**

The overall transfusion rate was 4.8 % (3.6 % for hip, 2.7 % for knee, and 15.0 % for spine surgery). Independent predictors of PRBC transfusion included: preoperative hemoglobin < 13 g.dL^-1^ (Relative Risk [RR] 6.55), high-risk surgical procedures (RR = 7.40), ASA physical status III‒IV (RR = 2.00), absence of tranexamic acid use (RR = 2.52), and, to a lesser extent, age > 75 years (RR = 1.50). The combination of all identified risk factors was associated with a markedly increased transfusion risk (RR = 14.55; *p* < 0.0001).

**Conclusion:**

These findings have informed modifications to our clinical practice, aimed at enhancing quality standards through the implementation of more effective Patient Blood Management (PBM) strategies.

## Introduction

Major Orthopedic Surgery (MOS) often requires allogeneic Blood Transfusion (BT), which is associated with various complications,^1^ including transmission of infectious diseases, venous thromboembolism, pulmonary embolism, myocardial infarction,^2^ transfusion-related acute lung injury, increased risk of surgical site infection,^2^, ^3^, ^4^ prolonged hospital stay,^2^ and elevated morbidity and mortality rates.^5^

The implementation of blood-sparing strategies, optimization of Patient Blood Management (PBM) protocols,^6^ routine use of Tranexamic Acid (TXA),^7^ advancements in surgical techniques, and updated transfusion thresholds and clinical guidelines^8^^,^^9^ have all contributed to a decline in transfusion rates in recent years. Concurrently, increasing life expectancy and the demand for higher healthcare standards have led to a growing volume of major orthopedic procedures. The primary goal of elective orthopedic surgery is to restore patients’ functional status without increasing the incidence of postoperative complications.^10^ However, patient-specific characteristics must always be considered in perioperative planning.^11^

The first 24 hours following surgery represent a critical window for determining the ultimate surgical outcome. During this period, the extent of bleeding and the requirement for transfusion are key factors, given the potential for coexisting complications such as hemodynamic instability, oliguria, impaired tissue perfusion, and acidosis, along with the inherent risks of transfusion.

Several specific characteristics of our center are relevant to this study:

- Our hospital is located in a rural area, 16 km from the nearest tertiary care center.
- It is a specialized, elective orthopedic surgery facility with dedicated teams for knee, hip, and spine procedures.
- There is no emergency department or trauma admissions (e.g., fractures).
- Staffing and laboratory resources are limited, particularly during on-call shifts.
- Due to these constraints, patients remain in the Post-Anesthesia Care Unit (PACU) for enhanced monitoring throughout the day of surgery.
- The anesthesia department maintains a prospectively updated database that records all clinical events occurring in the PACU during the first 24 postoperative hours.
- Hospital policy regarding the preoperative reservation of Packed Red Blood Cells (PRBC) for orthopedic procedures has been revised to be more restrictive.

Given these contextual factors, the primary objective of this study is to identify independent Preoperative Risk Factors for PRBC Transfusion (PRF-PRBC-T) within the first 24 hours following MOS. Identifying these predictors will support informed preoperative counseling and enable the implementation of targeted strategies aimed at reducing transfusion rates and improving perioperative outcomes.

### Study design and methods

This was a six-year observational study with retrospective follow-up, including a total of 7072 patients who underwent surgery between March 2018 and April 2024. The study was approved by the Clinical Research Ethics Committee of Navarra on April 28, 2023 (Registry n° PI_2023/32), and by the Managing Director of the University Hospital of Navarra on May 2, 2023 (Registry n° 465).

Inclusion criteria encompassed the following procedures: Total Knee Arthroplasty (TKA), TKA Revision (TKAR), Total Hip Arthroplasty (THA), THA Revision (THAR), Spine Arthrodesis (SA), and Scoliosis Surgery (SS).

Exclusion criteria included amputations, tumor-related surgeries, and major upper limb procedures.

All data were obtained from a prospectively maintained institutional database with no missing values. The variables analyzed included: age, sex, weight, height, Body Mass Index (BMI), American Society of Anesthesiologists (ASA) physical status classification, type of surgery, preoperative and postoperative hemoglobin values (measured at 8:00 AM on the first postoperative day), use of Tranexamic Acid (TXA), and requirement for PRBC transfusion.

The TXA using protocol during the study period was: hip and knee surgery 10 mg.kg^-1^ IV and 2.5 g locally infiltrated; spine surgery initial bolus of 10 mg.kg^-1^ IV and infusion of 1 mg.kg^-1^.h^-1^ during surgery. The use of TXA in the PACU was at the discretion of the attending physician.

Based on institutional experience and unpublished internal data, TKAR, THAR, and SS were classified as high-risk procedures for transfusion (more than 10 % of patients transfused). Our historical transfusion rates were: TKAR 10.79 %, THAR 12.70 %, and SS 69.81 %.

### Statistics

Normality of data distribution was assessed using histograms and the Kolmogorov-Smirnov test. Homogeneity of variances was evaluated with Levene’s test. Continuous variables are presented as mean ± Standard Deviation (SD) and 95 % Confidence Interval (95 % CI). Between-group differences were assessed using Student’s *t*-test for normally distributed data, or the Mann-Whitney *U* and Kruskal-Wallis tests for non-normally distributed data. The unequal variance *t*-test (Welch *t* Test) was used rather than Student's *t*-test when the sample sizes for each group differed (significant different variances).

Categorical variables are reported as relative frequencies (percentages) and ranges. Group comparisons for categorical variables were conducted using Pearson’s chi-squared test or Fisher’s exact test, as appropriate.

Correlation between continuous variables was assessed using Pearson’s correlation coefficient; for ordinal or non-normally distributed variables, Spearman’s correlation coefficient was used.

Univariate logistic regression was performed to identify potential predictors of blood transfusion. Variables with *p* < 0.10 in univariate analysis were included in a multivariate logistic regression model (forward stepwise) to identify independent predictors and control for confounding. Model significance was assessed using the omnibus test of model coefficients, and goodness of fit was evaluated using the Hosmer-Lemeshow test. Predictive performance was further assessed using Receiver Operating Characteristic (ROC) curve analysis.

Youden’s J statistic and Odds Ratios (ORs) with 95 % Confidence Intervals (95 % CIs) were calculated to determine optimal threshold values for sensitivity and specificity in distinguishing transfused versus non-transfused patients.

Preoperative hemoglobin levels were recoded using the Youden index-derived cutoff value to calculate Relative Risks (RRs) via the MedCalc online tool (https://www.medcalc.org/calc/).

All analyses were performed using SPSS version 26.0 (IBM Corp., Armonk, NY, USA). Statistical significance was set at a two-tailed *p*-value of 0.05.

## Results

Preoperative values and corresponding transfusion rates by surgical group and procedure type are presented in Table 1, Table 2, respectively.

**Table 1:** Preoperative characteristics by surgical group (hip, knee, and spine).

	Hip	Knee	Spine	Total	*p*
**n**	2737	3344	991	7072	
**%**	38.7	47.3	14.0	100	
**Age**	66.2 ± 11.8 [65.8 – 66.7]	70.3 ± 8.6 [70.0 – 70.6]	57.7 ± 17.2 [56.6 – 58.9]	67.0 ± 12.1 [66.7 – 67.3]	< 0.0001
**Sex**					< 0.0001
Male	59.9	49.7	45.5	53.1	
Female	40.1	50.3	54.5	46.9	
**Weight**	78.9 ± 15.7 [78.1 – 79.4]	81.2 ± 15.1 [80.7 – 81.8]	75.4 ± 16.8 [74.4 – 76.6]	79.5 ± 15.7 [79.1 – 79.9]	< 0.0001
**Height**	166.0 ± 9.7 [165.6 – 166.4]	163.2 ± 9.5 [162.9 – 163.6]	164.4 ± 10.0 [163.9 – 165.2]	164.4 ± 9.7 [164.2 – 164.7]	< 0.0001
**BMI**	28.5 ± 4.8 [28.3 – 28.7]	30.4 ± 5.1 [30.3 – 30.6]	27.8 ± 5.4 [27.4 – 28.2]	29.3 ± 5.1 [29.2 – 29.5]	0.009
**BMI class**					< 0.0001
UW	0.6	0.1	4.5	0.9	
HW	22.5	11.9	24.6	17.7	
OW	42.2	38.6	38.6	40.0	
O-I	25.7	32.2	23.3	28.4	
O-II	6.9	12.3	7.0	9.5	
O-III	2.0	4.9	2.1	3.4	
**ASA**	2.3 ± 0.6 [2.3 – 2.4]	2.5 ± 0.6 [2.5–2.6]	2.3 ± 0.7 [2.3 – 2.4]	2.4 ± 0.6 [2.4 – 2.5]	< 0.0001
I	8.6	2.9	11.1	6.3	
II	47.7	45.1	44.8	46.1	
III	39.9	48.7	40.6	44.1	
IV	3.8	3.4	3.6	3.5	
**Hb**	14.4 ± 1.3 [14.4 – 14.5]	14.2 ± 1.2 [14.2 – 14.3]	13.8 ± 1.7 [13.8 – 14.0]	14.2 ± 1.3 [14.2 – 14.3]	< 0.0001
**Hb**					
> 13	87.1	85.3	74.8	84.6	< 0.0001
< 13	12.9	14.7	25.2	15.4	< 0.0001
< 12	3.0	3.2	11.3	4.3	< 0.0001
< 11	0.8	0.9	6.3	1.5	< 0.0001
< 10	0.3	0.3	3.7	0.8	< 0.0001
**TXA use**	87.1	82.4	85.2	84.6	< 0. 0001

Age (yr), weight (kg), height (cm), Body Mass Index (BMI, kg.m^-2^), ASA, American Society of Anesthesiologists physical status category) and preoperative Hemoglobin (Hb) (g.dL^-1^) are expressed as mean ± standard deviation.

Continuous variables ‒ age (years), weight (kg), height (cm), Body Mass Index (BMI, kg·m⁻²), ASA physical status classification, and preoperative hemoglobin (Hb, g.dL⁻¹) ‒ are presented as mean ± standard deviation and [95 % Confidence Interval].

Categorical variables ‒ surgical group (hip, knee, spine), sex, BMI classification according to the World Health Organization [Underweight (UW), Healthy Weight (HW), Overweight (OW), Class I Obesity (O-I), Class II Obesity (O-II), Class III Obesity (O-III)], ASA classification, preoperative hemoglobin categories (< 13 to < 10 g·dL⁻¹), and Tranexamic Acid (TXA) use ‒ are expressed as percentages.

**Table 2:** Preoperative characteristics by type of surgery.

	THA	THAR	TKA	TKAR	SA	SS	*p*
**n**	2549	188	2962	382	885	106	
**%**	36.0	2.7	41.9	5.4	12.5	1.5	
**Age**	66.1 ± 11.7 [65.7 – 66.6]	67.7 ± 13.1 [65.7 – 69.7]	70.1 ± 8.6 [69.8 – 70.5]	71.6 ± 9.0 [70.7 – 72.6]	60.8 ± 13.1 [59.9 – 61.7]	32.4 ± 24.6 [25.6 – 31.6]	< 0.0001
**Sex**							
Male	59.9	60.6	48.9	55.6	48.4	21.7	
Female	40.1	39.4	51.1	44.4	51.6	78.3	< 0.0001
**Weight**	78.8 ± 15.7 [78.2 – 79.5]	77.4 ± 15.4 [75.0 – 79.8]	81.2 ± 15.1 [80.7 – 81.8]	81.1 ± 14.8 [79.6 – 82.7]	78.0 ± 15.5 [76.9 – 79.1]	54.2 ± 10.7 [51.5 – 55.8]	< 0.0001
**Height**	166.1 ± 9.7 [165.7 – 166.5]	164.2 ± 9.5 [162.8 – 165.7]	163.2 ± 9.5 [162.9 – 163.6]	163.2 ± 9.5 [162.2 – 164.2]	164.8 ± 10.0 [164.2 – 165.6]	160.8 ± 9.4 [159.6 – 163.5]	< 0.0001
**BMI**	28.5 ± 4.8 [28.3 – 28.7]	28.5 ± 4.5 [27.8 – 29.2]	30.4 ± 5.1 [30.2 – 30.7]	30.4 ± 5.1 [29.9 – 31.0]	28.6 ± 4.9 [28.3 – 29.0]	21.1 ± 4.7 [19.7 – 21.6]	< 0.0001
**BMI class**							< 0.0001
UW	0.6	0.6	0.6	0.0	0.6	36.8	
HW	22.9	17.8	17.8	13.1	21.8	47.4	
OW	42.0	44.8	44.8	38.0	41.9	10.5	
O-I	25.4	30.1	30.1	30.2	25.7	3.2	
O-II	7.0	5.5	5.5	13.7	7.5	2.1	
O-III	2.1	1.2	4.9	5.0	2.4	0.0	
**ASA**	2.3 ± 0.6 [2.3 – 2.4]	2.6 ± 0.7 [2.5 – 2.7]	2.5 ± 0.6 [2.4 – 2.5]	2.6 ± 0.6 [2.6 – 2.7]	2.4 ± 0.6 [2.4 – 2.5]	2.0 ± 0.9 [1.8 – 2.2]	< 0.0001
I	8.9	4.9	3.0	2.1	7.4	41.5	
II	48.7	34.8	46.3	35.3	47.4	22.6	
III	39.2	49.5	47.5	57.4	41.6	32.1	
IV	3.2	10.9	3.1	5.3	3.5	3.8	
**Hb**	14.4 ± 1.2 [14.4 – 14.5]	13.7 ± 1.6 [13.5 – 14.0]	14.3 ± 1.2 [14.3 – 14.4]	13.9 ± 1.5 [13.7 – 14.0]	14.0 ± 1.5 [14.0 – 14.2]	12.0 ± 2.3 [11.4 – 12.5]	< 0.0001
**Hb**							
> 13	88.2	73.4	86.4	77.0	78.4	44.3	< 0.0001
< 13	11.8	26.6	13.6	23.0	21.6	55.7	< 0.0001
< 12	2.2	13.3	2.5	8.4	8.0	38.7	< 0.0001
< 11	0.4	5.9	0.4	3.4	3.6	28.3	< 0.0001
< 10	0.2	1.6	0.1	1.6	1.9	18.9	< 0.0001
**TXA use**	88.1	72.1	83.4	74.3	84.8	89.6	< 0.0001

Continuous variables ‒ age (years), weight (kg), height (cm), Body Mass Index (BMI, kg·m⁻²), ASA physical status classification, and preoperative hemoglobin (Hb, g.dL⁻¹) ‒ are expressed as mean ± standard deviation and [95 % Confidence interval].

Categorical variables ‒ including surgical procedures [Total Hip Arthroplasty (THA), Total Hip Arthroplasty Revision (THAR), Total Knee Arthroplasty (TKA), Total Knee Arthroplasty Revision (TKAR), Spine Arthrodesis (SA), and Scoliosis Surgery (SS)], sex, BMI classification according to the World Health Organization [Underweight (UW), Healthy Weight (HW), Overweight (OW), Class I Obesity (O-I), Class II Obesity (O-II), Class III Obesity (O-III)], ASA classification, hemoglobin categories (< 13 to < 10 g.dL⁻¹), and tranexamic acid (TXA) use ‒ are expressed as percentages.

The mean first-day hemoglobin loss, defined as the difference between preoperative hemoglobin and hemoglobin measured at 8:00 AM on postoperative day 1, was −2.9 ± 1.0 g.dL^-1^. Significant differences were observed among surgical groups: hip −2.8 ± 1.0, knee −2.9 ± 0.9, and spine −3.2 ± 1.3 g.dL^-1^ (*p* < 0.0001), as well as among specific procedures: THA −2.8 ± 1.0, THAR −3.0 ± 1.3, TKA −2.8 ± 0.8, TKAR −3.4 ± 1.1, SA −3.2 ± 1.2, and SS −3.2 ± 2.3 g.dL^-1^ (*p* < 0.0001). Notably, scoliosis surgery patients often receive transfusions on the first postoperative day, which likely underestimates true hemoglobin loss in this group.

The mean first-day hemoglobin loss was −2.9 ± 1.0 g.dL^-1^ for both men and women. Hemoglobin loss was similar between sexes for hip (−2.8 ± 1.0 vs. −2.9 ± 1.1 g.dL^-1^) and knee surgeries (−3.0 ± 1.0 vs. −2.9 ± 0.9 g.dL^-1^) but differed in spine surgery (−3.4 ± 1.3 vs. −3.1 ± 1.4 g.dL^-1^). Stratifying by procedure and sex, hemoglobin loss was comparable for THA (−2.8 ± 1.0 g.dL^-1^ in both sexes), THAR (−3.0 ± 1.2 vs. −3.0 ± 1.4), and TKA (−2.9 ± 0.9 vs. −2.8 ± 0.9), while men experienced greater hemoglobin loss in TKAR (−3.6 ± 1.2 vs. −3.2 ± 1.0), SA (−3.4 ± 1.2 vs. −3.2 ± 3.0), and SS (−4.2 ± 2.5 vs. −3.0 ± 2.2 g.dL^-1^).

Table 3 summarizes demographic characteristics and hemoglobin values for transfused and non-transfused patients. Significant correlations with transfusion requirement were found for sex, weight, height, BMI, BMI category, type of surgery, preoperative hemoglobin, hemoglobin thresholds (< 13, < 12, < 11, and < 10 g.dL^-1^), use of Tranexamic Acid (TXA), and high-risk surgical classification (all *p* < 0.0001, Spearman's rho).

**Table 3:** Demographic and preoperative hemoglobin characteristics by transfusion status.

	Transfused	Non-transfused	*p*
**n**	337	6735	
**%**	4.8	95.2	
**Age**	62.0 ± 23.3 [58.7 – 64.4]	67.1 ± 11.4 [67.0 – 67.6]	< 0.0001
**Sex**			< 0.0001
Male	3.1	96.9	
Female	6.7	93.3	
**Weight**	68.2 ± 16.2 [66.0 – 69.9]	79.9 ± 15.3 [79.6 – 80.4]	< 0.0001
**Height**	160.4 ± 9.6 [159.2 – 161.6]	164.6 ± 9.6 [164.4 – 164.9]	< 0.0001
**BMI**	26.5 ± 5.9 [25.7 – 27.1]	29.4 ± 5.0 [29.3 – 29.6]	< 0.0001
**BMI class**			< 0.0001
UW	10.5	0.5	
HW	28.5	17.3	
OW	35.7	40.2	
O-I	16.6	29.0	
O-II	6.9	9.6	
O-III	1.8	3.5	
**ASA**	2.6 ± 0.8 [2.6 – 2.8]	2.4 ± 0.6 [2.4 – 2.5]	< 0.0001
I	11.9	6	
II	23.5	47.2	
III	51.5	43.8	
IV	13.1	3.1	
**Hb**	12.4 ± 1.8 [12.2 – 12.7]	14.3 ± 1.3 [14.3 – 14.4]	< 0.0001
**Hb**			
> 13	4.8	95.2	< 0.0001
< 13	16.9	83.1	< 0.0001
< 12	34.2	65.8	< 0.0001
< 11	49.1	50.9	< 0.0001
< 10	55.6	44.4	< 0.0001
**TXA use**	3.5	96.5	< 0.0001
**Hip**	3.6	96.4	< 0.0001
THA	2.9	97.1	
THAR	13.8	86.2	
**Knee**	2.7	97.3	< 0.0001
TKA	1.5	98.5	
TKAR	11.5	88.5	
**Spine**	15.0	85.0	< 0.0001
SA	8.0	92	
SS	73.6	26.4	

Continuous variables ‒ age (years), weight (kg), height (cm), Body Mass Index (BMI, kg.m⁻²), ASA physical status classification, and preoperative hemoglobin (Hb, g·dL⁻¹) ‒ are expressed as mean ± standard deviation and [95 % Confidence interval].

Categorical variables ‒ including type of surgery (hip, knee, or spine), sex, BMI classification based on the World Health Organization [Underweight (UW), Healthy Weight (HW), Overweight (OW), Class I Obesity (O-I), Class II Obesity (O-II), Class III Obesity (O-III)], ASA classification, hemoglobin categories (< 13 to < 10 g·dL⁻¹), Tranexamic Acid (TXA) use, and transfusion rates ‒ are reported as percentages.

A binary logistic regression using the enter method identified potential predictors of transfusion. Variables with *p* < 0.10 (age *p* = 0.014, sex *p* = 0.029, ASA class *p* < 0.0001, surgical group *p* < 0.0001, procedure type *p* < 0.0001, preoperative hemoglobin *p* < 0.0001, TXA use *p* < 0.0001, and high-risk surgical category *p* < 0.0001) were included in a multivariate logistic regression using forward stepwise (likelihood ratio) selection (Table 4). In the final model, sex was excluded as an independent predictor (*p* = 0.424).

**Table 4:** Binary logistic regression analysis using the stepwise forward likelihood ratio method to identify independent predictors of blood transfusion.

	β	SE β	Wald’s χ^2^	df	Wald test *p*-value	OR	95 % CI	
							Inferior	Superior
**Age**	0.013	0.006	5.411	1	0.020	1.013	1.002	1.025
**ASA**	−0.415	0.119	12.127	1	< 0.0001	0.661	0.523	0.834
**Group**	0.710	0.088	64.799	1	< 0.0001	2.033	1.711	2.417
**Surgery**	−0.676	0.069	94.864	1	< 0.0001	0.509	0.444	0.583
**Hb**	0.747	0.054	192.816	1	< 0.0001	2.110	1.899	2.345
**TXA**	−0.793	0.171	21.448	1	< 0.0001	0.453	0.324	0.633
**High risk**	1.481	0.155	91.113	1	< 0.0001	4.399	3.245	5.963
**Constant predictor**	−7.639	0.874	76.359	1	< 0.0001	0.000	NA	NA
**Omnibus test of model coefficients**			744.085	7	< 0.0001			
**Goodness-of-fit test: Hosmer-Lemeshow test**			7.979	8	0.436			

Model performance is indicated by Cox and Snell R² = 0.110; Nagelkerke R² = 0.374.

Regression outputs include: β (regression coefficient), SE β (standard error of β), df (degrees of freedom), OR (Odds Ratio), and 95 % CI (95 % Confidence Interval).

Predictors analyzed: age, ASA classification (American Society of Anesthesiologists physical status), surgical group (hip, knee, or spine), specific surgical procedure [Total Hip Arthroplasty (THA), Total Hip Arthroplasty Revision (THAR), Total Knee Arthroplasty (TKA), Total Knee Arthroplasty Revision (TKAR), Spine Arthrodesis (SA), Scoliosis Surgery (SS)], preoperative Hemoglobin (Hb), Tranexamic Acid (TXA) use, and classification as high-risk surgery for transfusion.

**Figure 1:**
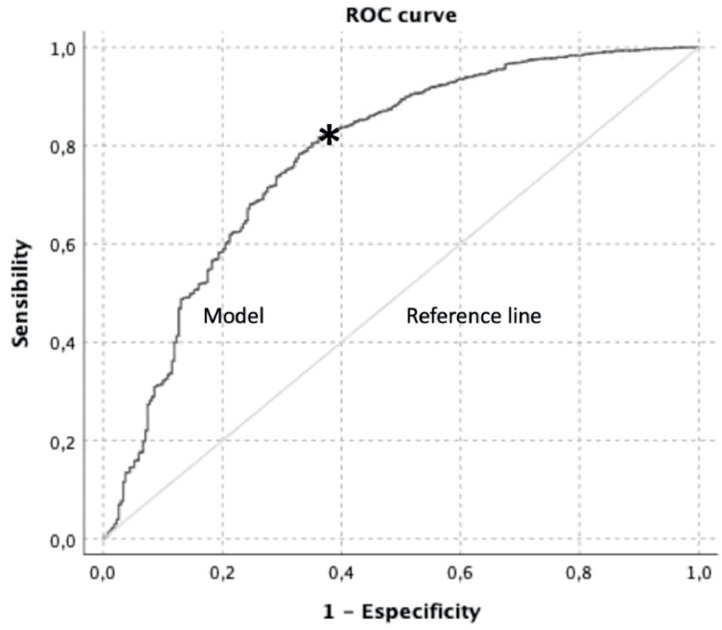
Receiver Operating Characteristic (ROC) curve for the Table 4 full model as predictor of blood transfusion.

**Figure 2:**
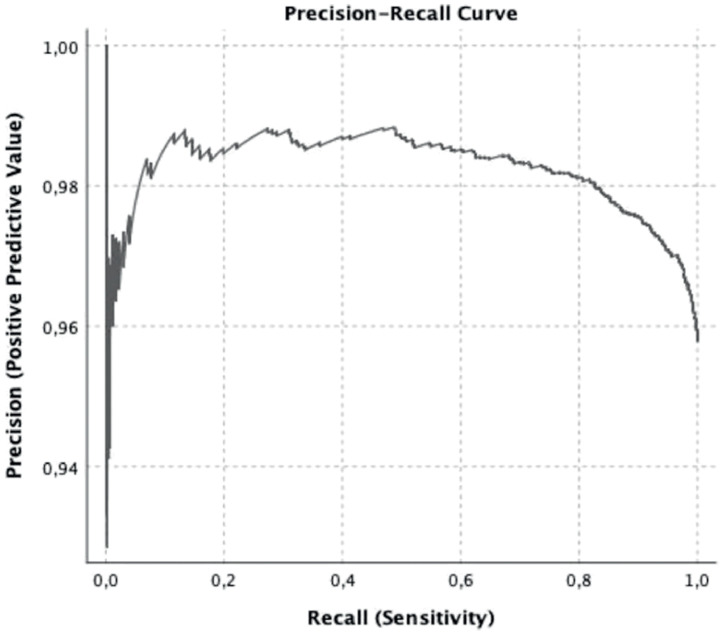
Precision-recall curve of the model of the Table 4 full model as predictor of blood transfusion.

Receiver Operating Characteristic (ROC) curve analyses were conducted to evaluate predictive accuracy and determine optimal cutoff points via Youden’s index. The full model exposed in Table 4 had an overall quality of 0.75 (Youden’s index = 0.456; AUC = 0.779 ± 0.016, *p* < 0.0001, 95 % CI 0.747–0.811; ROC curve in Figure 1 and precision-recall curve in Figure 2). The Brier score of the full model was 0.03, indicating that the probabilistic prediction model is very good, since it predicts probabilities that closely match the observed outcomes. The Events-Per-Variable (EPV) ratio was 48, well above the recommended minimum of 10, indicating that the model was robust and the estimates for each predictor were reliable.

The variable with the highest predictive value was preoperative hemoglobin (Youden’s index = 0.49; AUC 0.817 ± 0.014, *p* < 0.0001, 95 % CI 0.790–0.844). This was followed by procedure type (Youden’s index = 0.65; AUC 0.689 ± 0.020, *p* < 0.0001, 95 % CI 0.650–0.729) and classification as high-risk surgery (Youden’s index = 0.65; AUC 0.688 ± 0.020, *p* < 0.0001, 95 % CI 0.649–0.726). Lower predictive values were observed for ASA classification (Youden’s index = 0.55; AUC 0.591 ± 0.020, *p* < 0.0001, 95 % CI 0.551–0.631), TXA use (Youden’s index = 0.54; AUC 0.582 ± 0.019, *p* < 0.0001, 95 % CI 0.544–0.620), and age (Youden’s index = 0.47; AUC 0.503 ± 0.019, *p* = 0.862, 95 % CI 0.465–0.541).

The most significant PRF-PRBC-T are related either to patient characteristics, namely, preoperative hemoglobin < 13.15 g.dL^-1^, ASA class III or higher and age > 75.5 years ‒ or to surgical factors, such as undergoing a procedure with high transfusion risk or having contraindications to TXA use.

Preoperative hemoglobin < 13.15 g.dL^-1^ (cutoff per ROC, Youden’s index = 0.490, RR = 6.55, *p* < 0.0001 and OR = 7.59, *p* < 0.0001) demonstrated the highest predictive power for transfusion, with a ROC curve showing excellent discriminative ability.^12^

The optimal ASA class cutoff value was 2.5 (Youden’s index = 0.178), corresponding to the threshold between ASA II and ASA III. Patients classified as ASA III or IV had a transfusion RR = 2.00 (*p* < 0.0001) and OR = 2.07 (*p* < 0.0001).

Age also showed a significant association, with a cutoff of > 75.5 years (Youden’s index = 0.084) yielding an RR of 1.50 (*p* = 0.0002) and OR = 1.54 (*p* = 0.0002).

The presence of all three demographic risk factors (hemoglobin < 13.15 g.dL^-1^, ASA > III and age > 75.5 years) conferred a cumulative transfusion RR of 4.45 (*p* < 0.0001) and OR = 5.18 (*p* < 0.0001).

Regarding surgical factors, patients undergoing a high-risk procedure had an RR of 7.40 (*p* < 0.0001) and OR = 9.21 (*p* < 0.0001), while those not receiving TXA had an RR of 2.52 (*p* < 0.0001) and OR = 2.67 (*p* < 0.0001). The combination of both surgical risk factors resulted in a transfusion RR of 6.13 (*p* < 0.0001) and OR = 7.87 (*p* < 0.0001).

When all five risk factors ‒ both demographic and surgical ‒ were present, the cumulative risk of transfusion increased markedly, with a relative risk of 14.55 (*p* < 0.0001) and OR = 38.27 (*p* < 0.0001).

## Discussion

BT is associated with a range of postoperative complications in MOS,^1^ including increased risk of wound infection, venous thromboembolism, pulmonary embolism, myocardial infarction, and prolonged hospital stay ‒ particularly in spine fusion surgery (*n* = 13,695).^2^ Therefore, identifying Preoperative Risk Factors for Packed Red Blood Cell Transfusion (PRF-PRBC-T) is essential for improving preoperative patient stratification, enabling inclusion in Patient Blood Management (PBM) programs, and offering personalized risk information.

Moreover, recognizing PRF-PRBC-T contributes to reducing the healthcare system's burden by decreasing postoperative complications and optimizing resource utilization. This includes tailoring PBM interventions, rationalizing preoperative crossmatching, considering autologous blood donation, implementing perioperative blood salvage systems,^13^ and limiting postoperative hemoglobin testing to patients at higher risk.^14^

Ideally, PRF-PRBC-T should be identifiable during preoperative evaluation through patient history, physical examination, and standard blood tests. We evaluated age, sex, height, weight, BMI, ASA physical status, hemoglobin concentration, surgery type, and TXA use.

Advanced age (≥ 60 years) has been widely recognized as a transfusion risk factor in MOS,^2^^,^^14^, ^15^, ^16^, ^17^, ^18^ while in pediatric spinal fusion, younger age has been associated with increased risk.^19^ In our cohort, the mean age of transfused patients (62.0 years) was paradoxically lower than that of non-transfused patients (67.1 years), due to the younger age distribution of the scoliosis surgery group, which also had the highest transfusion rate. Nevertheless, age > 75.5 years was independently associated with increased transfusion risk.

Several studies have identified female sex as a risk factor for BT in orthopedic procedures^18^ including TKA,^20^ Total Joint Arthroplasty (TJA = TKA + THA),^17^^,^^21^ and pediatric spine fusion.^19^ However, sex was not an independent predictor in our analysis and in another study.^16^ While women had higher transfusion rates (Table 3), this was likely due to lower baseline hemoglobin levels (13.6 ± 1.1 g.dL^-1^ in women vs. 14.8 ± 1.2 g.dL^-1^ in men), despite similar hemoglobin declines postoperatively (mean drop: −2.9 ± 1.0 g.dL^-1^).

The influence of weight, height, and BMI on transfusion risk remains controversial. Low body weight and low BMI have been reported as PRF-PRBC-T,^15^^,^^16^^,^^19^^,^^22^ whereas high BMI has been associated with reduced transfusion rates in TJA,^17^^,^^23^ and some studies have shown no association.^24^ In our cohort, BMI was not an independent risk factor.

Although race has been identified as a PRF-PRBC-T in children undergoing spinal fusion,^19^ it was not analyzed in our study due to the homogeneity of our patient population (Southern European).

Comorbidity burden, as reflected by ASA classification, has consistently been shown to predict transfusion risk across orthopedic procedures.^2^^,^^17^, ^18^, ^19^, ^20^ In our study, ASA class III or higher was independently associated with increased transfusion risk.

Surgical complexity ‒ such as revision TJA, multilevel spine fusion, scoliosis correction, and tumor resections ‒ is another well-known determinant of transfusion risk.^2^^,^^16^^,^^18^^,^^19^^,^^25^^,^^26^

PBM strategies target three main pillars ‒ optimizing erythropoiesis, minimizing blood loss, and improving physiologic tolerance to anemia ‒ across the pre-, intra-, and postoperative periods. We focused on preoperative variables and one intraoperative factor (TXA use). All patients had routine preoperative lab tests. Low hemoglobin was a strong predictor of transfusion, in line with previous studies.^14^, ^15^, ^16^^,^^18^^,^^19^^,^^21^^,^^22^^,^^25^, ^26^, ^27^ In fact, hemoglobin and age are often cited as the two most important predictors.^14^^,^^18^ However, we argue that surgical type and ASA classification can, in some cases, outweigh the impact of age. Notably, our cutoff for age (75.5 years) was higher than in prior studies, where thresholds of 60 years were more common.^15^

Numerous studies have proposed a hemoglobin threshold of 13 g.dL^-1^ to reduce transfusion risk. Some recommend 12 g.dL^-1^ as the minimum for THA.^28^ Regardless of the exact value, higher hemoglobin levels are consistently associated with lower transfusion rates.^17^ Although the transfusion threshold is commonly set at 7 g.dL^-1^, real-world decisions must consider broader clinical context, especially in older patients with comorbidities and acute bleeding.

In our study, the optimal preoperative hemoglobin cutoff for predicting transfusion was 13.15 g.dL^-1^, which closely aligns with the widely accepted minimum of 13 g.dL^-1^. The average hemoglobin loss within the first 24 hours postoperatively was approximately 3 g.dL^-1^, underscoring the clinical relevance of this threshold. Patients with preoperative hemoglobin < 13 g.dL^-1^ had significantly higher transfusion rates (Table 3).

At our institution, a perioperative PBM program has substantially reduced transfusion rates. Patients with preoperative hemoglobin < 13 g.dL^-1^ undergo automated iron studies, and their lab results are reviewed by Internal Medicine for appropriate management with iron, folate, erythropoietin, or combination therapy. In our country, anemia is detected in 6.6 % of patients scheduled for arthroplasty, with 14.5 % having suboptimal hemoglobin (< 13 g.dL^-1^), and 32.4 % having iron deficiency.^29^ In our series, 15.4 % had suboptimal hemoglobin, highlighting the opportunity for preoperative optimization.

Regarding intraoperative blood conservation, TXA has proven effective in reducing blood loss in MOS (*n* = 4921),^7^ and is recommended for all patients undergoing TJA regardless of preoperative hemoglobin.^30^ Although TXA use is often planned in advance, individual clinical decisions may evolve intraoperatively.

Given the high prevalence and clinical burden of MOS, identifying transfusion risk factors is essential for guiding clinical decisions and improving patient outcomes. Our study has several strengths: it includes a large sample size (*n* = 7072), uses prospectively updated perioperative data, and focuses on transfusion risk within the first 24 hours postoperatively ‒ a period associated with significant hemodynamic instability and adverse outcomes.

However, the study has limitations. It was conducted at a specialized orthopedic hospital with dedicated surgical teams, which may not reflect transfusion rates in general hospitals. Still, our center treats complex referral cases, often at higher risk of bleeding. Furthermore, our findings may not be generalizable to non-Southern European populations due to differences in demographics and baseline hemoglobin levels.

This study has several strengths and limitations that warrant discussion. Although retrospective in nature, it is based on data extracted from a continuously updated database that records multiple perioperative variables in real time. Therefore, it is not merely a review of potentially incomplete medical records. As a result, no cases were missed in our analysis. Moreover, the large sample size (*n* = 7072) provides substantial statistical power for validation and enhances the generalizability of the findings.

Geographic location ‒ used here as a proxy for population ethnicity^19^ ‒ is another factor to consider when extrapolating these results to other populations. In this context, it is important to highlight that this study contributes valuable data from a Southern European population, complementing the existing literature, which predominantly focuses on Asian cohorts.

The study was conducted in a specialized orthopedic surgery hospital, where all staff members are exclusively dedicated to orthopedic procedures. This may result in lower transfusion rates compared to general hospitals. However, it should also be noted that, as a referral center, this institution often handles more complex surgical cases, which are inherently associated with a higher risk of bleeding.

An important limitation to acknowledge is that this study only addresses transfusion risk within the first 24 hours postoperatively. Although some patients may require transfusion later during their hospital stay, there is a paucity of data focusing specifically on the immediate postoperative period ‒ when the greatest hemodynamic compromise typically occurs. This represents an added value of our study, particularly given that patients undergoing MOS are often elderly and present with comorbidities that limit their physiological reserve and tolerance to acute blood loss.

We are currently operating in a clinical landscape where patients increasingly demand both comprehensive information about procedural risks and optimal levels of postoperative well-being outcomes ‒ sometimes with unrealistic expectations. It is imperative, therefore, to provide the most accurate and individualized risk assessments possible. Patients should be informed of their specific risks based on their physical status and the nature of the planned procedure, including the likelihood of requiring a blood transfusion.

Clinical data must be systematically collected and analyzed in accordance with evidence-based medicine principles to generate reliable conclusions that can guide improvements in care quality. Understanding these data enables the implementation of perioperative strategies aimed at reducing transfusion requirements and postoperative complications in high-risk patients. Those identified with multiple transfusion risk factors should undergo thorough preoperative assessment and optimization to minimize avoidable and unnecessary risks.

## Conclusion

Preoperative hemoglobin < 13 g·dL^−1^, high-risk surgical procedures, ASA class III–IV, absence of TXA use, and age > 75 years are independent predictors of transfusion risk within the first 24 hours after major orthopedic surgery.

Identifying these factors preoperatively allows for targeted PBM interventions and improved perioperative planning. Given demographic and healthcare system variability, conducting local studies is advisable to guide context-specific clinical practice.

## Data availability statement

The datasets generated and/or analyzed during the current study are available from the corresponding author upon reasonable request.

## Authors’ contributions

All authors contributed equally to the conception, design, data analysis, drafting, and critical revision of the manuscript. All authors have read and approved the final version. The authors affirm that they meet the criteria for authorship as defined by the International Committee of Medical Journal Editors.

## Funding and resources

The authors report no external funding or sponsor involvement that could have influenced the outcomes of this research.

## Conflicts of interest

The authors declare no conflicts of interest.
